# Non-Contact Characterization of TPA-like Texture Properties of Gel-Based Soft Foods Using a Controlled Airflow–Laser System

**DOI:** 10.3390/foods15071166

**Published:** 2026-03-30

**Authors:** Hui Yu, Shi Yu, Meng He, Xiuying Tang

**Affiliations:** College of Engineering, China Agricultural University, No. 17 Qinghua East Road, Beijing 100083, China; b20223070587@cau.edu.cn (H.Y.); s20233071461@cau.edu.cn (S.Y.); hemengiii@cau.edu.cn (M.H.)

**Keywords:** non-contact and nondestructive detection, airflow–laser coupling, TPA-like measurement, adaptive Savitzky–Golay smoothing, soft food texture characterization

## Abstract

Texture characteristics are critical quality evaluation indicators for soft foods. Traditional texture profile analysis (TPA) relies on probe–sample contact and may cause irreversible structural damage, limiting its application in nondestructive or online detection. In this study, a non-contact and nondestructive Controlled Airflow–Laser Texturemeter (CAFLT) system was developed to achieve rapid multi-parameter texture characterization. The system integrates programmable airflow loading with laser displacement sensing to implement a TPA-like double-cycle loading protocol, simultaneously acquiring time–applied airflow pressure (T–AP) and time–displacement (T–D) responses. Gelatin–maltose composite gels with graded Bloom strengths (CL50–CL250) were used as model samples. Texture-related descriptors were extracted using a dual-curve feature framework and compared with traditional TPA measurements. The CAFLT system produced a double-peak response pattern resembling that of traditional TPA and showed clear monotonic trends with increasing gel strength. Hardness_CAFLT exhibited a strong correlation with the reference TPA hardness value (r = 0.97). In addition, Gumminess_CAFLT showed a positive association with traditional gumminess (r = 0.87), but should be interpreted within the CAFLT-specific loading framework. Multivariate principal coordinates analysis further demonstrated clear multivariate discrimination among samples. Additionally, the time-domain descriptor *t*_Peak1_ showed a strong power-law relationship with Bloom strength ($R^{2}=0.96$), indicating enhanced sensitivity to mechanical differences under small-deformation conditions. Overall, the CAFLT system provides a feasible approach for non-contact, nondestructive, and quantitative texture evaluation of soft foods, and shows strong potential for real-time quality monitoring and intelligent food inspection.

## 1. Introduction

The texture characteristics of food are one of the core attributes influencing consumer acceptance and sensory preferences. Their precise and objective quantitative characterization holds significant scientific importance and engineering value for food quality control, new product development, and processing technology optimization [1,2]. Currently, texture evaluation in the food industry typically integrates two approaches: manual sensory evaluation and instrumental measurement [3,4]. Among these, texture profile analysis (TPA), which is an instrumental method based on double-compression testing, can quantify key parameters such as hardness, springiness, resilience, and cohesiveness. It has been widely adopted and is regarded as one of the standard methods for the objective evaluation of food texture [5,6]. In contrast, sensory evaluation can comprehensively reflect multi-dimensional sensory information, its inherent subjectivity, individual variability, and lack of repeatability severely limit its application in standardization and online detection scenarios [7].

However, as the food industry evolves toward higher value-added, refined, and intelligent manufacturing, the inherent limitations of traditional contact-based TPA technology have become increasingly unavoidable in practical applications. The primary concern is that the direct mechanical contact between the probe and the sample during testing inevitably causes irreversible structural damage [8,9], making it difficult to meet the demands for nondestructive testing and continuous quality assessment in high-value-added or high-hygiene-requirements products, such as gummy candies, functional gels, and dairy-based gels [9,10,11]. Previous studies have demonstrated that in the measurement of adhesiveness of high-sugar or high-viscosity systems, the adhesion/de-adhesion process itself can introduce significant methodological measurement errors [12,13]. Furthermore, the presence of sugar crystals or granules at the interface can substantially alter the measurement of adhesive force and work of adhesion, thereby increasing the uncertainty and risk of bias in texture parameter calculations [14]. These issues severely restrict the sustainable application of traditional TPA in scenarios involving continuous inspection and online quality monitoring.

To overcome the inherent limitations of contact-based detection, non-contact methods for characterizing food texture and material viscoelasticity have gained increasing attention in recent years. These include approaches based on visual imaging (e.g., machine vision and 3D surface reconstruction) [15,16,17], optical sensing (e.g., near-infrared and hyperspectral imaging) [17,18,19], ultrasonics (e.g., air-coupled ultrasound) [20,21], and airflow excitation [14,22]. Among these, the concept of non-contact mechanical testing, which combines airflow excitation with optical displacement measurement, has been preliminarily validated in the field of mechanical characterization of materials. For instance, Xu et al. proposed a controlled laser airflow detection (CLAFD) technique, which integrates airflow excitation with laser displacement measurement. This approach enables a comprehensive assessment of multiple mechanical properties under creep loading conditions, providing a methodological basis for the feasibility of air–laser-coupled methods [23]. In the field of food inspection, air jet impingement technology offers distinct advantages in avoiding physical contact damage by regulating airflow pressure for non-contact loading [11,24,25,26]. However, the existing research primarily focused on predicting individual texture indices (such as hardness/tenderness or gel strength) using single-impingement or creep modes [24,25,26]. It remains challenging to achieve a multi-parameter, full-profile texture characterization (e.g., springiness, cohesiveness, and chewiness) in a TPA-like manner. Meanwhile, unavoidable turbulence and impingement pressure fluctuations during the airflow loading process often introduce high-frequency signal noise [27]. This noise can obscure subtle deformation features on the sample surface, thereby reducing the signal-to-noise ratio and the reliability of parameter extraction. To some extent, these challenges have limited the further application of airflow excitation methods in TPA-like texture analysis.

It should be noted that the non-contact testing approach, which combines airflow excitation with optical displacement measurement, has clear applicability limitations. Accordingly, in this study, “gel-based soft foods” refer to soft, viscoelastic, deformable food-like solids that can undergo stable small deformation without fracture under non-contact airflow excitation (e.g., gel- and gummy-type products, as well as dairy gel systems) [28]. Very stiff or brittle materials, as well as extremely soft or fragile thin-layer systems that rupture or become unstable under airflow loading, are outside the scope of the current study. Compared to hard or brittle foods, soft and semi-solid foods typically exhibit more pronounced viscoelastic responses, generating resolvable deformation signals even under small external loads. Consequently, these materials are better suited for non-contact characterization using gentle excitation methods such as airflow [29,30]. Furthermore, gel-based foods (e.g., gummy candies, gelatin gels, and milk protein gels) have long been utilized as model systems for discussing and validating TPA methodologies due to their relatively homogeneous structures, adjustable formulations/network architectures, and high reproducibility of mechanical and textural parameters [6,31]. In addition, dairy gel systems are frequently characterized using instrumental tools like TPA during processing (e.g., coagulation or gelation) for process characterization and quality correlation analysis [32]. In this study, we focus on standardized gel-based gummy model systems as a representative soft-food category for controlled instrumental benchmarking. Therefore, selecting soft gel foods as research objects not only facilitates the feasibility validation of the non-contact airflow excitation method but also provides a rational experimental foundation for subsequent comparative evaluation of selected texture-related indicators against traditional TPA.

To this end, a non-contact Controlled Airflow–Laser Texturemeter (CAFLT) system was developed for TPA-like characterization of soft-food texture under non-contact conditions. The system integrates a programmable pneumatic loading module with a high-precision laser displacement sensing module. By constructing a quasi-static ramp loading–unloading cycle under fully non-contact conditions, it simulates the double-compression process utilized in traditional TPA. To address signal noise caused by airflow turbulence, an adaptive Savitzky–Golay smoothing-based signal processing strategy was also proposed to enhance the stability and interpretability of the airflow pressure–displacement response signals. At this stage, we focus on instrumental feasibility and benchmarking using standardized gel models. Sensory validation is beyond the scope of the present study and will be addressed in future work using real edible products. This study was conducted based on the following central hypothesis: the time-domain mechanical characteristics of the airflow pressure–displacement response obtained under controlled airflow loading exhibit interpretable relationships with selected texture-related attributes measured by traditional TPA. From a mechanical perspective, the peak applied airflow pressure reflects the overall resistance of the sample to external deformation and corresponds to the hardness parameter in TPA, whereas the rebound displacement and the integral features during the loading–unloading process are related to recovery- and response-associated mechanical behaviors of the material. Therefore, non-contact airflow excitation is expected to enable TPA-like multi-parameter texture characterization without causing structural damage to the sample.

To verify this hypothesis, composite gel-based gummy model samples with different Bloom strength levels were employed as test materials. The study systematically carried out the following tasks: (1) the construction of the hardware and software architecture of the CAFLT system and the establishment of the testing protocol; (2) the validation of an adaptive signal-processing strategy to improve the stability and interpretability of airflow pressure–displacement response signals; and (3) a comprehensive evaluation of the statistical comparability and discrimination capability of the CAFLT system compared to traditional TPA through both single-parameter correlation analysis and multivariate statistical methods (e.g., principal coordinates analysis). The study provides a feasible technical approach for non-contact and nondestructive testing of soft food texture characteristics, offering methodological references for real-time quality monitoring and online intelligent detection in future food industry production lines.

## 2. Detection Principles and Methodology

### 2.1. Non-Contact TPA-like Detection Principle

**Figure 1:**
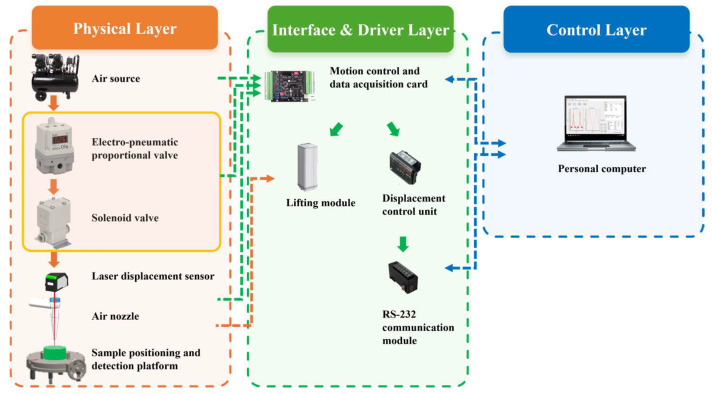
Schematic diagram of the non-contact CAFLT detection system for soft foods.

The working principle of the non-contact Controlled Airflow–Laser Texturemeter (CAFLT) system for soft foods is illustrated in Figure 1. The system is based on the synchronized coupling of air jet impingement loading and laser triangulation displacement measurement, and mainly consists of four functional modules: a master control unit, an airflow excitation module, a displacement sensing module, and a mechanical positioning module.

The core concept of the proposed detection system is to employ a controllable airflow pressure load to simulate the mechanical compression applied by the probe of a traditional texture analyzer, thereby enabling non-contact and nondestructive loading of samples. Similar non-contact airflow excitation approaches have been explored in food mechanics and rheological characterization. For example, the gas-pulse-based Foodtexture Puff Device enables quantitative evaluation of sample adhesiveness and mechanical responses through non-contact airflow loading combined with displacement measurement [24]. In addition, studies on air jet impingement in food processing have provided a physical basis for understanding airflow–material interactions [33]. Building upon these previous efforts, the present study integrates non-contact airflow excitation with a controllable loading strategy to develop the CAFLT system for non-contact TPA-like measurements.

During the preparation stage, the mechanical positioning module adjusts the height of the detection head to establish a standardized stand-off distance between the nozzle and the sample surface. Once testing begins, the master control unit generates linearly modulated control signals according to a predefined TPA-like timing sequence. These electrical signals are converted by the airflow excitation module into an airflow jet with linearly increasing pressure, which is vertically applied to the sample surface to produce a non-contact distributed pressure load.

Meanwhile, the displacement sensing module employs optical triangulation to capture minute surface deformations at the loading center in real time with a high sampling rate. A closed-loop control strategy based on displacement feedback and pressure cut-off is implemented. When the measured deformation reaches the preset target displacement, the control unit immediately reverses the loading signal, driving the airflow to unload linearly at the same rate, thereby completing a single loading–unloading cycle.

By executing the standard TPA sequence of first compression–relaxation–second compression, the system simultaneously records the excitation signal (pressure-related) and the response signal (deformation-related), generating synchronized time–airflow pressure (T–AP) and time–displacement (T–D) curves. Here, the airflow pressure refers to the applied pressure signal delivered by the proportional valve (expressed in kPa). Finally, characteristic features are extracted using dedicated non-contact texture analysis algorithms to achieve quantitative and nondestructive characterization of key texture attributes of soft foods, including hardness, springiness, and cohesiveness.

### 2.2. Basis of Linear Airflow Loading

Traditional TPA tests are typically performed under a displacement-controlled mode, in which the probe is driven into the sample at a constant speed while the corresponding mechanical force response is recorded during compression. In contrast, the CAFLT system proposed in this study operates based on airflow excitation and inherently follows an airflow pressure-programmed (stress-driven) loading mode, where the externally applied airflow pressure load is regulated as a function of time and the resulting deformation response of the sample is simultaneously monitored.

Under the assumption of small-strain linear viscoelasticity, stress-driven and strain-driven excitations exhibit a well-defined duality in rheological theory. According to linear viscoelastic principles and the Boltzmann superposition principle, the material response to an arbitrary time-dependent pressure input can be interpreted as the linear superposition of a series of infinitesimal instantaneous responses [34]. Therefore, within the small-deformation linear viscoelastic regime, pressure-controlled (stress-driven) and displacement-controlled loading approaches can provide related viscoelastic information for characterizing material response within the linear range [35,36]. Moreover, under dynamic loading conditions, the introduction of smooth ramp-type loading pulses has been shown to improve the uniformity of internal stress distribution and facilitate a more stable quasi-constant strain rate.

In the field of non-contact testing, previous studies have experimentally demonstrated the feasibility of airflow excitation for food materials. Bamelis et al. [11] and Morren et al. [24] reported that transient pneumatic pulses applied to food surfaces produced deformation responses that were associated with the storage modulus (G′) and loss modulus (G″) measured using traditional rheometers, indicating that non-contact airflow loading can effectively characterize the viscoelastic properties of food systems.

Based on these theoretical and experimental foundations, the CAFLT system adopts a control sequence of linear pressurization–displacement feedback cut-off–linear unloading. Although this loading pathway is mechanically opposite to that of traditional TPA, in that CAFLT applies a pressure-programmed (stress-driven) excitation whereas traditional TPA uses displacement-controlled (strain-driven) compression, the resulting pressure–displacement responses obtained under small deformation conditions may retain interpretable relationships with selected mechanical characteristics measured by traditional TPA. This provides a sound theoretical basis for non-contact extraction of TPA-like texture-related indicators under small-deformation conditions.

### 2.3. Signal Processing and Parameter Extraction

#### 2.3.1. Signal Preprocessing and Noise Reduction

Due to limitations in sensor precision, data acquisition quantization errors, and airflow-induced turbulence, the raw time–displacement signals inevitably contain occasional abrupt spikes as well as high-frequency noise components. To improve data quality and ensure reliable extraction of subsequent texture parameters, a systematic signal preprocessing workflow was established, consisting of two main steps: outlier removal and waveform smoothing.

First, abrupt signal fluctuations caused by signal loss or sensor abnormalities were identified and removed using the Local Outlier Factor (LOF) algorithm. Unlike simple threshold-based filtering, the LOF method is density-based and quantifies the abnormality of each data point by calculating the ratio between its local reachability density (LRD) and that of its k-nearest neighbors [37]. When the local density of a point is significantly lower than that of its neighboring points, resulting in an LOF value substantially greater than 1, the point is classified as an outlier and subsequently removed. The missing values generated after outlier removal were then filled using linear interpolation to restore temporal continuity and maintain the overall shape of the signal.

Subsequently, to further enhance signal quality, both fixed-window and adaptive-window Savitzky–Golay (SG) smoothing strategies were employed. Fixed-window SG smoothing (window length = 17) performs local polynomial fitting within a predefined window based on the least-squares principle [38]. This approach is effective for relatively stable signals; however, when noise distribution is uneven or signal variations are highly non-stationary, it may lead to either insufficient smoothing or excessive attenuation of critical features. To overcome these limitations, an adaptive-window SG smoothing approach was introduced. The core mechanism dynamically determines the optimal window length by constructing a composite error evaluation function based on local signal characteristics. Two optimization objectives were defined:

(i) To ensure that the smoothed displacement peaks of the first and second compressions (*DP*_1_ and *DP*_2_) closely approach the preset target displacement threshold, thereby better preserving the main deformation features; 

(ii) To minimize the absolute difference between the two peak values to maintain peak consistency between the two compression cycles.

Accordingly, a target deviation error (*E*_target_) and a peak discrepancy error (*E*_peak_) were defined, and a composite error metric (*E*_combined_) was constructed, as expressed in Equations (1)–(3):

(1) $$E_{target}=\frac{\left|{DP}_{1}-D_{target}|\;+\;|DP_{2}-D_{target}\right|}{2}$$

(2) $$E_{peak}=|DP_{1}-DP_{2}|$$

(3) $$E_{combined}=E_{target}+E_{peak}$$

where *DP*_1_ and *DP*_2_ denote the displacement peak values obtained during the first and second compression cycles after smoothing, respectively, while *D*_target_ represents the preset target deformation in the experiment (1.0 mm in this study). The algorithm searches odd-numbered window sizes within the range of 5–51 and determines the optimal smoothing parameter by minimizing *E*_combined_. This adaptive selection introduces both target-displacement constraints and peak-consistency constraints during the smoothing process, thereby improving signal consistency and providing a consistent data basis for subsequent feature extraction and analysis.

#### 2.3.2. Texture Feature Extraction Based on Dual Response Curves

**Figure 2:**
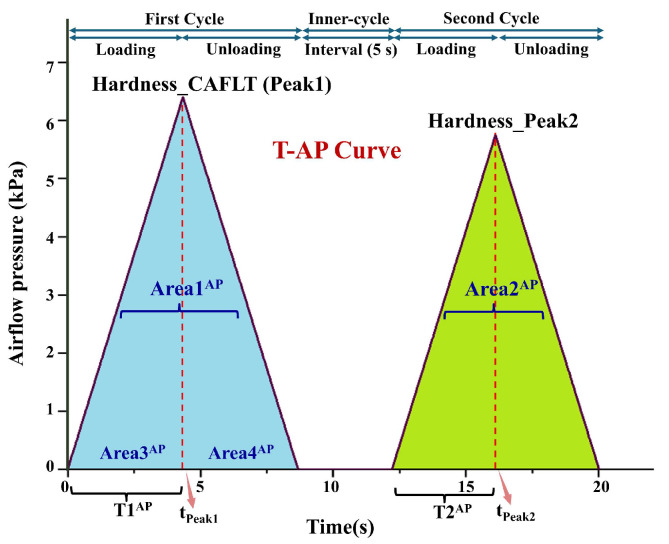
Typical time–airflow pressure (T–AP) response curve of a CAFLT-based TPA-like test. The first and second peak pressures are denoted as Peak1 and Peak2, respectively; Peak1 is used to define Hardness_CAFLT. *t*_Peak1_ indicates the time to reach Peak1. T1^AP^ and T2^AP^ denote the characteristic time intervals (defined on the T–AP curve) used to calculate Apparent Springiness_T–AP (T2^AP^/T1^AP^). The enclosed regions Area1–Area4 (superscript AP) are used for parameter extraction, and their definitions are summarized in Table 1. The top arrows indicate the loading/unloading stages of First Cycle and Second Cycle and the inter-cycle interval (5 s). Figure 2 and Figure 3 are plotted on the same time axis to facilitate direct comparison between excitation (T–AP) and deformation response (T–D).

**Figure 3:**
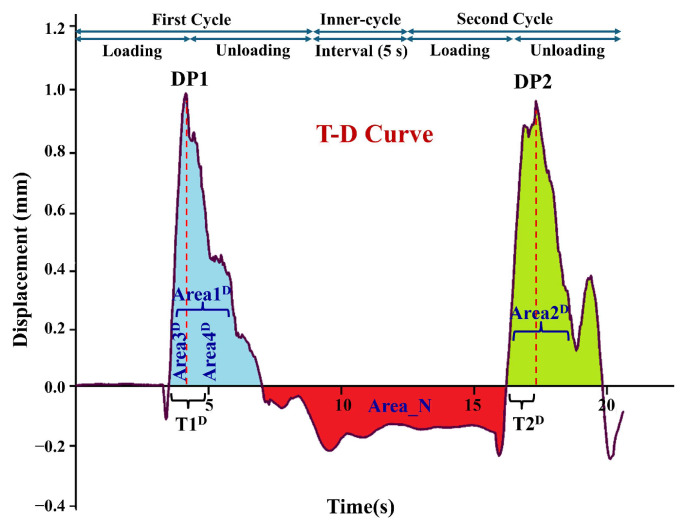
Typical time–displacement (T–D) response curve of a CAFLT-based TPA-like test. DP1 and DP2 denote the peak displacements (mm) in the first and second loading cycles, respectively. In each cycle, unloading is triggered when the preset target displacement is reached; Area_N indicates the negative area between the two cycles on the T–D curve, which is used to define Apparent Adhesiveness_T–D (see Table 1). Small negative displacement values may occur due to viscoelastic recoil and overshoot; the delayed response at the beginning of Cycle 2 reflects a time-lagged deformation response under airflow loading. The enclosed regions Area1–Area4 (superscript D) correspond to the areas used for T–D-based parameter extraction as defined in Table 1.

Unlike traditional contact-based TPA methods that primarily rely on a single mechanical response curve for parameter extraction, the proposed CAFLT system employs an airflow–laser coupled sensing strategy to enable dual-source synchronous monitoring of the testing process. The high-quality signals obtained after preprocessing (Section 2.3.1) were subsequently used to construct two characteristic response curves: a time–airflow pressure (T–AP) curve, representing the external airflow excitation intensity (Figure 2), and a time–displacement (T–D) curve, describing the deformation behavior of the sample (Figure 3).

**Table 1:** Definitions and Physical Meanings of CAFLT-Derived Texture Parameters.

Parameter	Definition	Meaning
Initial effective pressure, *P*_init_ (kPa)	Airflow pressure at the early loading stage corresponding to the time point at which the displacement signal first enters a sustained and monotonically increasing region on the T–AP curve.	Represents the minimum airflow pressure threshold required for the sample surface to transition from vibration or noise response to measurable and cumulative deformation, reflecting the initial mechanical sensitivity of the sample to external loading.
Hardness_ CAFLT (kPa)	Peak airflow pressure during the first compression stage of the T–AP curve (Peak1)	Indicates the resistance of the sample to airflow-induced deformation. A larger value corresponds to a harder texture and greater driving stress required to produce the same deformation.
Time to Peak1_T–AP, *t*_Peak1_(s)	Time point at which the peak airflow pressure (Hardness_CAFLT) is reached during the first compression stage of the T–AP curve.	Characterizes the time scale required for the sample to reach maximum resistance or target deformation under airflow loading. Larger values indicate slower deformation response and higher structural stiffness.
Resilience_ T-AP	Ratio of the unloading area of the second compression (Area4^AP^) to the loading area of the first compression (Area3^AP^) on the T–AP curve (Area4^AP^/Area3^AP^).	Describes the efficiency of energy recovery during airflow unloading. Values closer to 1 indicate synchronous unloading and recovery behavior, whereas lower values suggest greater viscous dissipation or hysteresis in the T–AP response.
Cohesiveness_ T-AP	Ratio of the second compression peak area (Area2^AP^) to the first compression peak area (Area1^AP^) on the T–AP curve (Area2^AP^/Area1^AP^).	Reflects the relative retention of the T–AP peak-area response after the first airflow impact and the ability to sustain repeated loading under CAFLT conditions.
Apparent Springiness_ T-AP	Ratio of the time required to reach the maximum pressure (Peak2) during the second compression to that of the first compression on the T–AP curve (T2^AP^/T1^AP^).	Represents the relative change in response time to reach the target deformation under repeated airflow loading. This loading-dependent metric reflects dynamic response speed and retention capability rather than true geometric recovery, and differs from traditional springiness.
Apparent “Adhesiveness”_ T-D (mm·s)	Negative displacement area (Area_N) on the T–D curve observed between the two compression cycles.	Reflects post-unloading dynamic lag or oscillatory overshoot in the displacement response. Because the protocol is non-contact, this metric does not represent true interfacial adhesion and is reported only as a descriptive indicator of surface response behavior.
Resilience_ T-D	Ratio of the unloading area of the second compression (Area4^D^) to the loading area of the first compression (Area3^D^) on the T–D curve (Area4^D^/Area3^D^).	Indicates instantaneous geometric recovery ability and elastic energy release after a single airflow impact. Lower values correspond to higher viscous loss in the displacement response.
Springiness_ T-D	Ratio of the time required to reach the target displacement during the second compression to that of the first compression on the T–D. curve (T2^D^/T1^D^).	Standard geometric springiness index representing the ability to recover to the same deformation under repeated compression, similar to the displacement-based definition in traditional TPA.
Gumminess_ CAFLT (kPa)	Product of Hardness_CAFLT and Cohesiveness_T–AP (Peak1 × Area2^AP^/Area1^AP^).	Comprehensive strength-related composite index for semi-solid foods, reflecting the combined effect of resistance to deformation and structural retention under CAFLT loading. This CAFLT-derived definition should be interpreted as a composite strength-related indicator rather than a strict one-to-one equivalent of traditional gumminess.

Note: Gelatin–maltose composite gummy samples prepared in this study exhibit a semi-solid viscoelastic gel structure [39]. In accordance with the classical texture classification framework [40,41] and subsequent methodological clarification [42], gumminess was selected as the representative parameter rather than chewiness, considering the nondestructive small-deformation regime employed in the CAFLT system. Some CAFLT-derived quantities, especially those related to cohesiveness, springiness, adhesiveness, and gumminess, should be interpreted as CAFLT-specific indicators under pressure-programmed non-contact loading rather than as strict one-to-one equivalents of the corresponding traditional TPA parameters.

Considering that gel-based gummies and other soft foods exhibit pronounced viscoelastic response, the pressure (stress) excitation and the corresponding strain development (deformation response) during linear airflow loading are not fully synchronized. A noticeable time lag and response difference are often observed between these two responses. Therefore, relying solely on a single mechanical response curve is insufficient to comprehensively characterize the mechanical response of the sample, particularly for capturing delayed recovery behavior and time-dependent deformation features.

To address this limitation, and with traditional probe-based (contact-based) TPA as the reference method (TPA_trad), a dual-curve feature extraction strategy was adopted in this study for CAFLT-based non-contact TPA-like measurement (TPA-like_CAFLT). Texture-related descriptors were independently calculated from the mechanical characteristics of the T–AP curve and the geometric characteristics of the T–D curve. The detailed definitions, symbols, and interpretations of the extracted quantities are summarized in Table 1. To distinguish the data sources and physical interpretations of different parameters, the notations T–AP and T–D were incorporated into the parameter names, where T–AP denotes pressure-based (airflow pressure–time) parameters derived from the applied airflow pressure response, and T–D denotes kinematic parameters derived from the displacement response. The subscript “CAFLT” indicates composite or comprehensive texture-related indices obtained using the proposed device.

## 3. Hardware and Software Design of the CAFLT System

### 3.1. Overall Hardware Architecture

**Figure 4:**
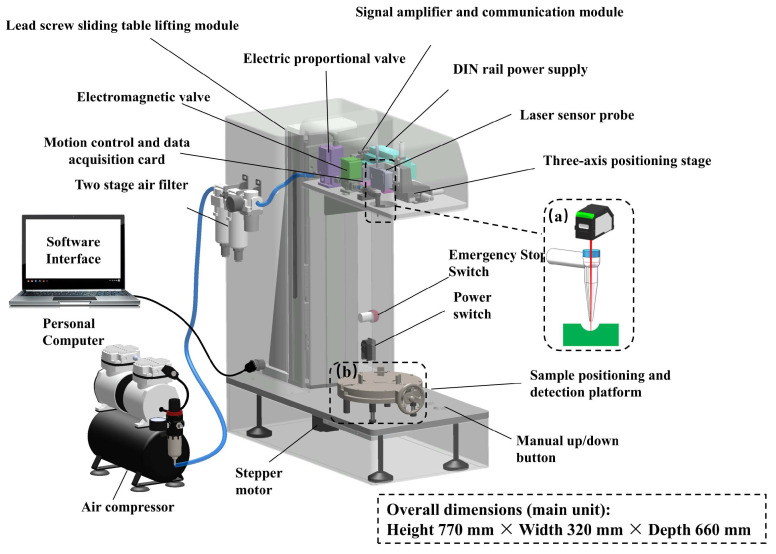
Hardware structure of the non-contact CAFLT detection device for soft foods: (**a**) Coaxial arrangement of the airflow nozzle and laser displacement sensor for vertical airflow loading and central displacement measurement; (**b**) Sample positioning and centering platform designed to improve positioning accuracy and test repeatability. The overall dimensions shown refer to the main unit only: 770 (H) × 320 (W) × 660 (D) mm (excluding external devices). The computer screen shows the software interface schematically; an enlarged view is presented in Figure 6.

The non-contact CAFLT detection system mainly consists of four functional modules: an airflow control unit, a displacement acquisition unit, a motion positioning unit, and a master control and data interface unit. The overall configuration of the system is illustrated in Figure 4. The system employs programmable airflow loading as the non-contact excitation source and laser displacement sensing for deformation measurement. Through a closed-loop control strategy, stable and repeatable loading–unloading cycles can be achieved for soft food samples.

The airflow control unit is responsible for generating a programmable pressure ramp excitation. The external air supply is first purified and pressure-stabilized before entering the electro-pneumatic proportional control module, enabling continuous regulation of the output airflow pressure. The modulated airflow is then delivered to the sample surface through a controlled actuation stage, forming a vertically distributed non-contact pressure load to simulate the compressive action applied by the probe of a traditional texture analyzer.

The displacement acquisition unit operates based on the principle of laser triangulation and monitors the surface deformation response of the sample in real time in a non-contact manner. To ensure high alignment between the loading axis and the measurement axis, the laser sensor and airflow nozzle are coaxially arranged in space, thereby guaranteeing that the loading direction coincides with the displacement measurement direction and minimizing lateral errors. The motion positioning unit is used to adjust the relative position between the detection head and the sample to establish a stable and repeatable stand-off distance ($S_{o}$), thus ensuring consistent testing conditions.

The master control and data interface unit performs system timing control and data communication functions. By synchronously acquiring the airflow excitation and displacement response signals, the system establishes a closed-loop control architecture centered on displacement feedback and displacement-triggered pressure cut-off (with unloading initiated once the target displacement is reached), providing a reliable hardware foundation for the stable execution of subsequent non-contact TPA-like testing procedures.

### 3.2. Quasi-Linear Airflow Loading Strategy

Considering that traditional TPA operates under a displacement-controlled mode, whereas the present CAFLT system employs airflow excitation and is driven by a programmed proportional valve-regulated line pressure (stress-driven) input, a quasi-linear voltage–pressure modulation airflow loading strategy was adopted to generate a quasi-static, transient triangular-ramp pressure waveform (with a zero-pressure interval between cycles) and to obtain loading-rate characteristics and viscoelastic response information that can be meaningfully compared with those achieved under constant-speed probe compression, while maintaining fully non-contact conditions. This strategy enables precise regulation of the airflow output.

Within the typical operating range of the system, the airflow loading intensity applied to the sample varies monotonically with the regulated line pressure. Therefore, by linearly modulating the input electrical signal of the proportional control module, a loading pattern in which the regulated pressure and thus the associated airflow-induced mechanical load vary approximately linearly with time can be achieved. Specifically, the system outputs a linearly increasing control signal to drive a gradual rise in airflow pressure, thereby generating a quasi-static quasi-linear mechanical loading excitation on the sample surface. In this study, the voltage ramp is linear by design, while the voltage–pressure and pressure–loading relationships are treated as quasi-linear within the calibrated operating range; therefore, the applied excitation is described as an approximately quasi-linear ramp-loading regime rather than a strictly linear loading input.

The loading process can be expressed by Equation (4):

(4) $$U_{out}\left(t\right)=\;k\cdot t+U_{0}$$

where

*U_out_* (*t*) is the control voltage signal applied to the proportional module at time;

*k* is the voltage ramp rate, which directly determines the loading rate of the airflow-induced pressure and is analogous to the probe descending speed in traditional TPA in terms of defining the loading time scale;

*t* denotes the loading time (s);

*U*_0_ is the initial bias voltage (V), used to compensate for the dead zone of the pneumatic system or to establish the initial loading condition.

Within the calibrated operating range, the regulated pressure follows the voltage ramp approximately linearly, and the airflow-induced loading intensity increases monotonically with pressure under fixed nozzle geometry and stand-off distance $S_{o}$. During testing, when the laser displacement sensor detects that the sample deformation reaches the preset target threshold, the control system immediately reverses the ramp in the loading signal, allowing the airflow pressure to enter a linear unloading stage at the same rate, thereby forming a complete loading–unloading cycle. By adjusting the loading-rate parameter *k*, the system can flexibly perform quasi-static tests under different loading-rate conditions. To further support the quasi-linear pressure-mapping assumption within the operating range, steady-state CFD-based calibration results are provided in the Supplementary Material (Figures S1 and S2). Figure S1 presents the outlet-plane total-pressure distributions under different inlet set pressures, while Figure S2 shows the corresponding regression between inlet set pressure and outlet-plane mean total pressure. Together, these results indicate that, under fixed nozzle geometry and $S_{o}$, the outlet-plane mean total pressure exhibits a highly stable and approximately linear dependence on the inlet set pressure within the operating range relevant to the present CAFLT experiments. Although the fitted line does not coincide with the identity line, the strong linearity demonstrates that the programmed inlet pressure can serve as a reliable and repeatable control variable for quasi-linear airflow loading.

**Figure 5:**
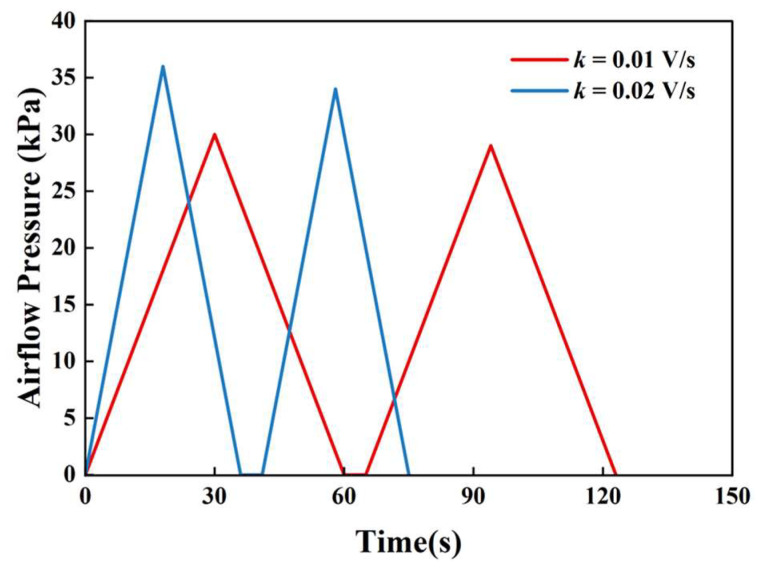
Schematic time–pressure curves under different loading rates k, illustrating the effect of loading rate on the time required to reach the same target displacement.

Different values of k correspond to different slopes of the regulated line airflow pressure ramp, as illustrated in Figure 5. A larger k results in a faster rise in airflow pressure, enabling the sample to reach the preset deformation within a shorter time. Therefore, parameter k plays a role analogous to the probe speed in traditional TPA tests and serves to regulate the loading-rate characteristics without altering the final target deformation. Although the compressibility of air may introduce slight nonlinearities, the resulting deviations from linearity were minimal within the operating range used in this study. Consequently, the overall loading process can be approximated as a stable, quasi-linear ramp-loading regime, satisfying the repeatability requirements of TPA-like measurements.

### 3.3. System Software Design and Development

**Figure 6:**
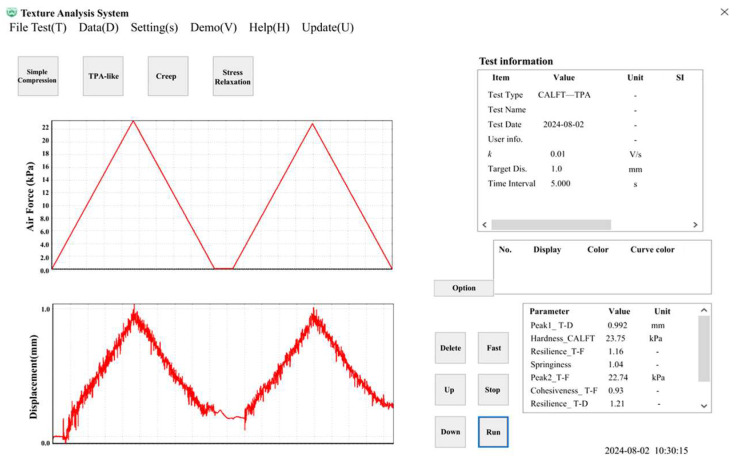
User interface of the host computer software for non-contact TPA-like detection. The interface includes modules for parameter configuration, real-time airflow pressure control, displacement monitoring, and data acquisition. The displayed airflow force and displacement curves were acquired under the TPA-like mode. The blue frame around the “Run” button indicates that the button was in the active state after the test was initiated.

To achieve precise control of the airflow excitation process and synchronized acquisition of multi-source signals, dedicated host-computer software was developed to enable parameter configuration, real-time control, and data acquisition and analysis. The graphical user interface is shown in Figure 6. The software adopts a modular architecture and mainly consists of four functional modules: parameter configuration, process control, data acquisition, and result analysis.

Prior to testing, key experimental parameters, including the loading rate, target deformation, and the interval between two compression cycles, can be preset through the software interface. Based on the defined settings, the system automatically generates the corresponding control sequence and synchronously drives both the airflow excitation module and the displacement acquisition module during operation. A built-in “displacement feedback–pressure cutoff” control logic continuously monitors the deformation response of the sample. Once the preset deformation threshold is reached, the system immediately switches to the unloading stage, thereby effectively preventing overloading and avoiding structural damage to soft food samples.

At the data acquisition level, the software enables time-synchronized recording of both the airflow excitation signal and the displacement response signal, and simultaneously generates dual-channel response curves, namely the time–airflow pressure (T–AP) and time–displacement (T–D) profiles, in real time. After completion of each test, the system automatically invokes the signal processing and parameter extraction modules to analyze the curve characteristics and calculate non-contact TPA-like texture discriptors, including hardness, springiness, and cohesiveness. This design establishes an automated closed-loop workflow that integrates hardware control with quantitative texture evaluation.

## 4. Materials and Methods

### 4.1. Experimental Materials and Sample Preparation

To avoid uncontrollable rheological variations introduced by formulation differences and batch-to-batch fluctuations in commercial gummy products, a standardized gelatin–maltose composite gel system was established in this study to prepare simplified gummy model samples, hereafter referred to as composite gel gummy model samples. Gelatin is a typical thermoreversible hydrogel whose three-dimensional network structure and viscoelastic properties can be precisely regulated by gel strength. Gel strength, commonly quantified by Bloom strength, is a key indicator for characterizing the texture properties of gummy and gel-based foods; therefore, gelatin has been widely employed as a model matrix in texture studies of soft foods. Sensory evaluation was not conducted in this stage because the gelatin–maltose samples were prepared as standardized model gels for controlled mechanical benchmarking (instrumental validation) rather than sensory-panel-ready edible products.

Ten types of gelatin powders with different nominal Bloom strengths were selected as the base materials, covering a broad range from low to high gel strength (CL50, CL80, CL100, CL120, CL140, CL160, CL180, CL200, CL220, and CL250), with CL50 sourced from Rousselot (Wenzhou) Gelatin Co., Ltd., Wenzhou, China, and the remaining samples sourced from Zhenhao Biotechnology Co., Ltd., Jinan, China. In all formulations, the gelatin concentration was kept constant, and only the nominal Bloom strength of the raw gelatin varied, thereby establishing a continuous gradient of mechanical properties within the composite gel system. Corn maltose syrup (Zhanyi brand, Shanghai Fengwei Industrial Co., Ltd., Shanghai, China) was incorporated as a filler and thickening component to simulate the modulation effect of the sugar phase on the gelatin network in industrial gummy products, and its proportion was maintained constant across all samples.

The preparation procedure of the composite gel gummy model samples was conducted with reference to the Chinese national standard GB 6783-2013 Food Additive Gelatin [43], with minor modifications according to experimental requirements. Specifically, a predetermined amount of gelatin powder was dispersed in distilled water to prepare a 6.67% (*w*/*v*) gelatin solution, followed by the addition of 10% (*w*/*v*) corn maltose syrup. The mixture was heated at 62 ± 3 °C using a temperature-controlled heating magnetic stirrer (DF-101S, Shanghai Licheng Bon West Instrument Technology Co., Ltd., Shanghai, China), until the gelatin was completely dissolved and the solution became homogeneous and transparent. The sol was then poured into cylindrical molds to prepare cylindrical gel samples with a diameter of 53 ± 1 mm and an effective height of 25 mm ± 1 mm. The mold dimensions used for preparation were 53 ± 1 mm in inner diameter and 32 ± 1 mm in height. The samples were allowed to cool naturally at room temperature and subsequently transferred to a 10 °C incubator (LHS-150, Yangzhou Peiyin Laboratory Instrument Co., Ltd., Yangzhou, China) for 18 h to complete gelation, yielding structurally stable and highly reproducible cylindrical gel samples.

To verify the validity and preparation reproducibility of the composite gel gummy model samples, the actual Bloom strength values of the ten sample groups were determined using the Bloom strength testing program integrated in the commercial texture analyzer (TA.XT Plus, Stable Micro Systems, Godalming, UK), with *n* = 5, and the statistical results are summarized in Table 2. The results showed that although slight systematic deviations existed between the measured and nominal Bloom strengths due to batch-to-batch variations in raw materials, all sample groups exhibited good preparation consistency. Except for the CL50 group, the coefficients of variation (CV) of the remaining nine groups were all below 3.1%, with an overall average CV of 2.09%, indicating high reproducibility of the sample preparation process. Furthermore, the measured Bloom strengths increased monotonically with the nominal Bloom grades (Figure S3). Linear regression performed on the grade means yielded R^2^ = 0.90 (Figure S3), indicating that the prepared samples formed a continuous Bloom-strength gradient across the selected nominal grades. This gradient system therefore provides a reliable model matrix for evaluating the performance of the non-contact CAFLT system.

### 4.2. Traditional TPA Measurements

Traditional TPA was performed as the reference method using the same commercial texture analyzer described above in Section 4.1. The testing procedure followed the standard protocol reported by Huang et al. [44], with minor adjustments according to the mechanical characteristics of the gel samples. All TPA measurements were performed in an air-conditioned laboratory with the thermostat set to 20 °C. After gelation at 10 °C, specimens were transferred to the laboratory environment and equilibrated at room temperature (approx. 20 °C) for 30 min prior to testing. Each measurement was initiated immediately after bench storage and completed within 5 min to minimize temperature drift.

During testing, an AOAC-recommended cylindrical probe (P/0.5R, 12.7 mm diameter, Delrin material; Stable Micro Systems, Godalming, UK) was employed. The prepared gelatin–maltose composite gel gummy samples were positioned directly beneath the probe and subjected to a typical double-compression mode. To ensure comparability among samples, the instrument was operated in force-trigger mode with a trigger force of 5 g, and a compressive strain of 75% was applied under displacement-controlled conditions to achieve the traditional large-deformation TPA test. The relaxation interval between the two compression cycles was set to 5 s [19,45,46]. For each Bloom strength level, five parallel measurements were conducted. Outliers (e.g., abnormal waveforms caused by probe slippage) were excluded, and the remaining results were averaged to obtain the final values. The detailed instrumental parameters are listed in Table 3.

### 4.3. Non-Contact TPA-like Measurements Using the CAFLT System

Full texture profile analysis of the gelatin–maltose composite gel gummy model samples was conducted using the self-developed non-contact CAFLT system established in this study. For clarity, texture parameters obtained from traditional probe-based TPA are denoted with the suffix _trad (e.g., Hardness_trad), whereas the TPA-like descriptors extracted from the CAFLT-based non-contact protocol are denoted with the suffix _CAFLT (e.g., Hardness_CAFLT). For CAFLT-specific descriptors derived from different waveforms, additional superscripts and labels are used to indicate the curve source (e.g., *^AP^ for the T–AP curve and *^D^ for the T–D curve), as summarized in Table 1. CAFLT measurements were performed under the same temperature condition and handling procedure as TPA_trad (Section 4.2; thermostat set to 20 °C, 30 min equilibration at ~20 °C prior to testing). Prior to testing, the airflow nozzle was mechanically adjusted to maintain a vertical stand-off distance $S_{o}$ of 10 mm above the sample surface to ensure consistent airflow loading conditions.

During testing, to obtain a loading process comparable in time scale to the quasi-static compression conditions of traditional TPA, a linear pressure–time loading strategy was adopted. Specifically, the airflow pressure was increased linearly via a proportional control module driven by a voltage ramp signal. The loading rate parameter *k* was defined as the voltage ramp rate applied to the proportional valve. In this study, the voltage loading rate was set to *k* = 0.01 V/s. Based on the calibration relationship between control voltage and output pressure, the pressure gain is 100 kPa/V, i.e., *p* = (100 kPa/V) × *U*, where *U* is the control voltage (V). Therefore, the pressure loading rate is:

(5) $$\frac{dP}{dt}=\left(100\frac{kPa}{V}\right)\times \frac{dU}{dt}=100k\;=\;1\;\mathrm{kPa}/s$$

Meanwhile, a laser displacement sensor continuously monitored the deformation response at the center of the sample in real time.

Considering that the present study focused on soft viscoelastic gel-based materials with relatively low airflow pressure levels, and to ensure appropriate engineering scale representation and improved readability, all airflow pressure values and related parameters are hereafter uniformly expressed in kPa throughout this study.

Regarding the target deformation setting, previous work by Yu et al. [7] demonstrated that small-deformation TPA tests (e.g., 4 mm compression) using a mechanical probe could effectively extract texture parameters and showed strong correlations with traditional large-deformation TPA results. Considering that the present study aimed to achieve fully non-contact and nondestructive texture characterization while avoiding any residual surface deformation or indentation after testing, a stricter micro-deformation control strategy was employed. The target deformation threshold for each compression was therefore set to 1.0 mm. This setting was intended to keep the samples within a small-deformation response regime and to minimize nonlinear disturbances induced by airflow loading under large deformations, thereby improving signal stability and interpretability.

When the laser sensor detected that the sample deformation reached the preset target value, the control system immediately switched to the unloading mode, allowing the airflow to decrease linearly at the same rate (1 kPa/s) until the airflow impact airflow pressure returned to zero. Under this control strategy, the loading and unloading processes formed a temporally symmetric mechanical waveform. Due to differences in sample hardness, the peak pressure and loading time required to reach the same target deformation varied dynamically among samples with different Bloom strength levels.

The two-cycle small-deformation protocol was designed to probe short-term recovery and within-sample repeatability in the viscoelastic regime. The compression–unloading cycle was repeated twice, with a relaxation interval (inter-cycle interval) of 5 s between the two loading cycles to provide a standardized short-term recovery window and to maintain comparability with commonly used traditional TPA settings before the second compression. Five parallel measurements were conducted for each gradient sample, and complete raw waveform data were recorded. Texture parameters were subsequently extracted and calculated according to the procedures described in Section 2.3.2. Although the inter-cycle interval in CAFLT was set to maintain a comparable short-term recovery window, the CAFLT protocol was not intended to reproduce fully identical test conditions to those of traditional TPA_trad, because it operates under pressure-programmed non-contact small-deformation loading rather than displacement-controlled large-deformation compression. Therefore, comparisons with TPA_trad in this study should be interpreted as benchmark-level evaluations of trend-level comparability and practical relevance, rather than strict one-to-one equivalence under matched test settings.

### 4.4. Data Processing

Each sample group was measured in five parallel replicates, and the results are expressed as mean ± standard deviation (mean ± SD). Raw data organization and preliminary processing were performed using Microsoft Excel, while traditional statistical analyses and graphical visualization were conducted using SPSS 26.0 and Origin 2021, respectively. All signal preprocessing and parameter extraction algorithms, including LOF-based outlier detection, fixed- and adaptive-window Savitzky–Golay smoothing, and area integration, were independently implemented on a Python platform (Python 3.10.9), primarily utilizing scientific computing libraries such as NumPy, SciPy, and pandas for data processing and feature extraction.

To evaluate the consistency and comparability between the CAFLT results and the reference method measurements, texture parameters obtained from the traditional texture analyzer were used as the benchmark. Pearson correlation analysis was employed to calculate correlation coefficients (r) between key traditional TPA parameters and TPA-like descriptors, thereby quantifying the degree of linear association between the two methods. Because the CAFLT and TPA_trad protocols were not conducted under fully identical test conditions, these analyses were intended to evaluate benchmark-level trend comparability and practical relevance rather than strict parameter equivalence under matched settings. Furthermore, to compare the capability of the two detection approaches in characterizing mechanical differences among samples at a multivariate level, a Bray–Curtis distance matrix was constructed based on the selected texture-related descriptors, and principal coordinate analysis (PCoA) was conducted to visualize multivariate relationships among samples in a reduced-dimensional space. To evaluate whether samples with different Bloom strength levels showed significant separation in the multivariate space, permutational multivariate analysis of variance (PERMANOVA) was performed using 999 permutations [47]. In PERMANOVA, the *p*-value (P) indicates the statistical significance of group separation based on permutations, whereas the PERMANOVA R^2^ (effect size) represents the proportion of variation in the distance matrix explained by the grouping factor. In addition, representative time–airflow pressure (T–AP) and time–displacement (T–D) response curves, along with three-dimensional waterfall plots, were generated to visually illustrate the dynamic loading–unloading behavior of different samples, providing a basis for subsequent texture parameter extraction and response interpretation.

## 5. Results and Discussion

### 5.1. Validation and Evaluation of the Signal Processing Algorithms

Although the CAFLT system proposed in this study eliminates the physical damage associated with contact-based probes, airflow impingement inevitably introduces high-frequency noise and occasional abrupt fluctuations due to turbulence disturbances. To validate the effectiveness of the signal processing workflow described in Section 2.3.1, the algorithm performance was quantitatively evaluated from two aspects: outlier removal and waveform smoothing.

#### 5.1.1. LOF Performance

**Figure 7:**
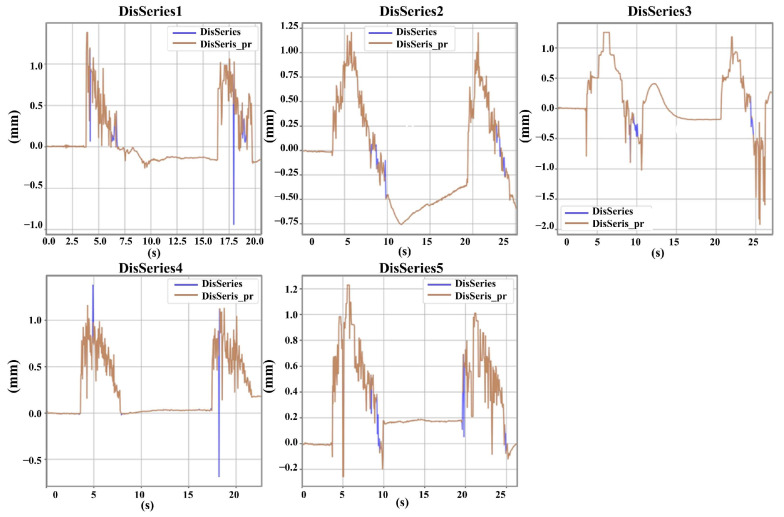
Visualization of LOF denoising effects on five parallel test curves of representative sample.

The Local Outlier Factor (LOF) algorithm was applied to the raw time–displacement signals to remove non-physical abrupt spikes caused by airflow turbulence or sensor blind zones. Figure 7 presents a comparison of representative signals before and after LOF processing. It can be observed that sharp outliers in the raw signals were effectively identified and eliminated, while the overall signal trend and local mechanical features were well preserved.

To quantitatively assess the denoising performance and signal fidelity of the LOF algorithm, the proportions of valid data, signal smoothness, and signal-to-noise ratio (SNR) before and after processing were statistically analyzed for ten gradient samples. The results are summarized in Table 4.

The statistical results showed that, after LOF processing, the proportion of valid data for each gradient sample decreased only slightly by approximately 1–2%, indicating that the algorithm removed only a small number of non-physical outliers caused by airflow turbulence or sensor anomalies, without significantly affecting the genuine mechanical response signals. Meanwhile, the overall smoothness index of the processed signals decreased, reflecting effective suppression of high-frequency noise. Variations in the signal-to-noise ratio (SNR) were observed among different samples, which may be attributed to differences in gel strength and airflow–sample interaction characteristics. In general, samples with low to medium Bloom strength exhibited relatively higher SNR values, whereas some medium-to-high-strength samples showed stronger fluctuations in the raw signals. Overall, the LOF preprocessing improved signal usability and stability without noticeably attenuating the signal energy, thereby providing a reliable data foundation for subsequent waveform smoothing and texture feature extraction.

#### 5.1.2. Performance Comparison of Fixed-Window and Adaptive Savitzky–Golay Smoothing

After outlier removal, the choice of smoothing strategy plays a critical role in the accurate extraction of texture-related features, particularly peak-related parameters such as hardness. Conventional Savitzky–Golay (SG) smoothing with a fixed window size (e.g., Window = 17) applies uniform filtering across the entire signal. While effective for stationary signals, this approach is often suboptimal for the non-stationary time–displacement responses generated under airflow excitation. In such cases, a fixed window may either insufficiently suppress high-frequency noise or excessively smooth peak regions, leading to biased estimation of deformation-related features.

**Figure 8:**
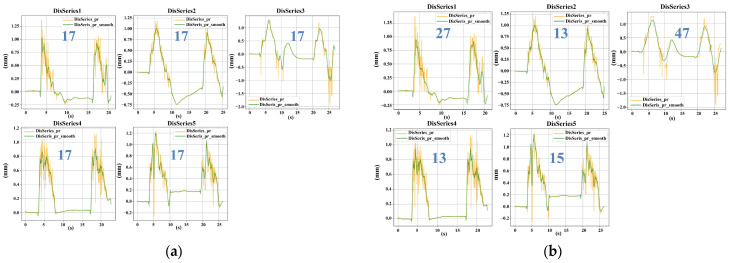
Comparison of smoothing effects between fixed-window and adaptive-window SG algorithms: (**a**) Results obtained using a fixed window (window length = 17); (**b**) Results obtained using the adaptive-window strategy. The yellow curves represent the displacement signals after LOF-based outlier removal (pre-smoothed signals), while the green curves denote the signals after Savitzky–Golay smoothing. The blue numbers in each subplot indicate the SG window length used for smoothing. In (**a**), the fixed window length was set to 17 for all signals; in (**b**), they denote the optimal window lengths automatically selected by the adaptive-window algorithm for each signal.

To address this limitation, an adaptive-window SG smoothing strategy was developed based on the minimization of a combined error metric *E_combined_*, which jointly considers the deviation of displacement peaks from the predefined target displacement and the consistency between the first and second compression responses. Figure 8 presents a visual comparison between the fixed-window SG method (Window = 17) and the proposed adaptive-window approach.

Unlike fixed-window smoothing, the adaptive strategy does not aim to strictly preserve the original waveform shape. Instead, it dynamically balances noise suppression and target displacement consistency by adjusting the window size according to local signal characteristics. Specifically, when high-frequency noise dominates or when the displacement peak deviates noticeably from the target displacement, a larger window is selected to suppress non-physical fluctuations. Conversely, in regions where the signal exhibits rapid but physically meaningful variations near the target displacement, the window size is reduced to avoid excessive smoothing. Therefore, the displacement peaks processed by the adaptive window may either be sharper or moderately flattened compared to the results of the fixed window. The core purpose is to make the displacement peaks obtained in the two compression processes approach the target deformation more stably and maintain good consistency.

The quantitative advantages of the adaptive-window strategy are summarized in Table 5. Across all gelatin samples, the adaptive approach consistently reduced the peak difference (Peak_Diff) and the combined error *E_combined_* relative to the fixed-window method. For example, in the CL80 sample, the fixed-window SG smoothing resulted in a high *E_combined_* value of 0.32, whereas the adaptive strategy, which automatically optimized the window size to approximately 31 with local adjustments, reduced *E_combined_* to 0.08. Similar improvements were observed across samples with different gel strengths.

In summary, the adaptive-window SG smoothing strategy demonstrated superior robustness under non-contact airflow excitation conditions. Rather than merely pursuing strict waveform preservation, this method prioritizes consistency with the target deformation and symmetry between compression cycles, thereby improving the reliability and comparability of texture feature extraction from non-stationary signals. Consequently, the adaptive-window SG smoothing algorithm was ultimately adopted as the unified preprocessing approach for all raw signals in the CAFLT system.

### 5.2. Typical Response Characteristics of the CAFLT System

#### 5.2.1. Morphological Comparison of Typical Response Curves

To establish the preliminary comparability between the output responses of the CAFLT system and those of traditional TPA measurements, this study first compared the two methods at the level of curve morphology. Specifically, the consistency between the traditional texture analyzer curves and the CAFLT curves was evaluated in terms of double-peak structure, temporal sequence of stages, and peak shape characteristics, thereby providing a foundation for subsequent equivalent parameter extraction and statistical comparisons.

**Figure 9:**
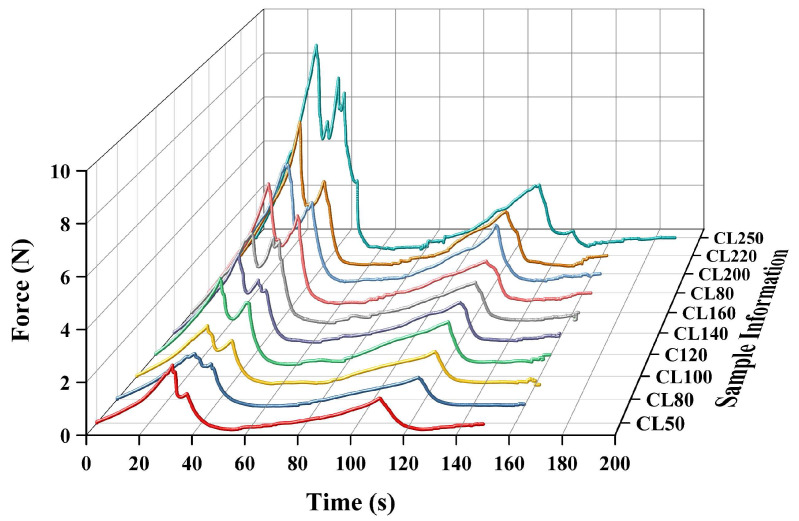
3D waterfall plot of time–airflow pressure (T–AP) responses of composite gel-based gummy model samples (CL50–CL250) measured using a traditional texture analyzer (TPA).

As shown in Figure 9, composite gel gummy model samples with different Bloom strength levels exhibited a typical time–airflow pressure (T–F) double-peak response under the traditional TPA mode. The first peak corresponded to the resistance of the sample to deformation during the initial compression stage, reflecting its hardness property; the near-zero airflow pressure region in the middle represented the relaxation period between the two compressions; and the second peak characterized the structural response of the sample during the second compression. The peak height and overall curve amplitude showed clear differentiation with increasing Bloom strength, indicating that the prepared composite gel gummy model samples possessed a stable texture gradient and good testing repeatability, and could therefore serve as a reliable reference system for validating the non-contact testing method.

**Figure 10:**
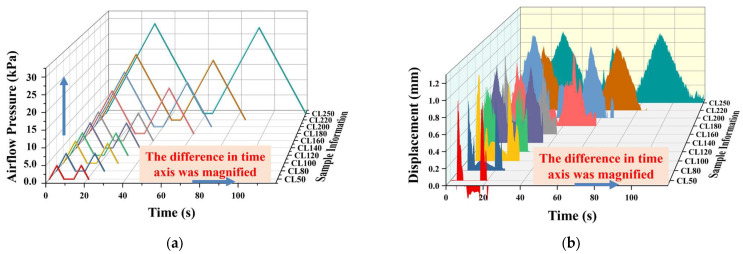
3D waterfall plots illustrating the evolution of dynamic responses of composite gel-based gummy model samples (CL50–CL250) measured using the CAFLT system: (**a**) time–airflow force (T–F) curves; (**b**) time–displacement (T–D) curves.From the overall trends observed in the three-dimensional waterfall plots, both types of response curves exhibited clear and continuous gradient variations with increasing gelatin Bloom strength, indicating that the CAFLT system can stably capture dynamic response differences arising from variations in the mechanical properties of the samples.

Based on this, the same samples with identical formulations and Bloom strengths were further tested using the non-contact CAFLT system, and the typical response curves obtained are presented in Figure 10. Specifically, Figure 10a shows the time–displacement (T–D) curve, which describes the deformation evolution of the sample during airflow loading and unloading, while Figure 10b shows the time–airflow pressure (T–AP) curve, reflecting the variation in external airflow excitation over time. It can be observed that both curves clearly exhibit two loading–unloading cycles corresponding to the first compression, relaxation stage, and second compression, thereby fully reproducing the fundamental mechanical sequence of the traditional TPA test.

Although the CAFLT system adopts an airflow pressure-controlled loading mode whereas traditional TPA employs displacement control, resulting in fundamentally different control paths, the overall morphological structures of the response curves remain highly consistent, both demonstrating the typical mechanical process of “loading–unloading–reloading.” This morphological correspondence indicates that the CAFLT system can effectively reconstruct the basic testing logic of traditional TPA under non-contact conditions, thereby providing a clear physical basis for extracting equivalent texture parameters from the dual T–AP and T–D curves in subsequent analyses.

#### 5.2.2. Dynamic Response Evolution of Composite Gel Gummy Model Samples Measured by CAFLT

To further evaluate the capability of the CAFLT system to discriminate soft foods with different mechanical strengths, ten groups of composite gel gummy model samples with continuous mechanical gradients (CL50–CL250) were tested. The overall evolution patterns of their time–airflow pressure (T–AP) and time–displacement (T–D) response curves are presented in Figure 10a and Figure 10b, respectively.

In the T–AP response curves (Figure 10a), owing to the constant-rate linear pressurization loading strategy adopted by the system, the airflow pressure increased linearly with time. Consequently, the differences among samples were primarily reflected in the peak pressure magnitude and the time required to reach the peak. As the Bloom value increased from CL50 to CL250, the first peak airflow pressure (Hardness_CAFLT) showed a monotonic increasing trend, rising from 4.53 kPa to 27.66 kPa, corresponding to an approximately 6-fold increase (Table 6). This result indicates that a denser gel network requires greater driving pressure to induce deformation under airflow loading, reflecting enhanced resistance to deformation and higher overall mechanical strength. Notably, this peak pressure represents macroscopic resistance during the effective deformation stage, whereas the initial deformation-triggering threshold has been independently characterized by the parameter *P*_init_. In contrast, the T–D response curves (Figure 10b) more intuitively revealed the temporal separation characteristics of the CAFLT system. With increasing sample hardness, the slope of the displacement curve gradually decreased, and the time required to reach the preset target deformation (1.0 mm) was markedly prolonged. A pronounced time-scale stretching was observed between extreme samples: CL50 reached the peak at approximately 4.53 s, whereas CL250 required up to 23.08 s, corresponding to a time difference exceeding 18 s (Table 6). This phenomenon suggests that differences in mechanical strength were effectively transformed into significant temporal response variations, producing a “time-domain amplification” effect that substantially enhances the discriminability among samples with different hardness levels.

The characteristic parameters extracted from the above response curves further confirmed this trend (Table 6). Among them, Hardness_CAFLT and Time to Peak1_T–AP exhibited the most pronounced and stable monotonic relationships with Bloom strength. In contrast, energy-related parameters, including Resilience, Cohesiveness, and Gumminess_CAFLT, as well as displacement-recovery-related parameters such as Apparent Adhesiveness, showed relatively small variations or higher fluctuations, mainly reflecting secondary structural behaviors under cyclic loading. Therefore, Time to Peak1_T–AP was selected as a representative temporal parameter to further characterize the time-amplification capability of the CAFLT system.

**Figure 11:**
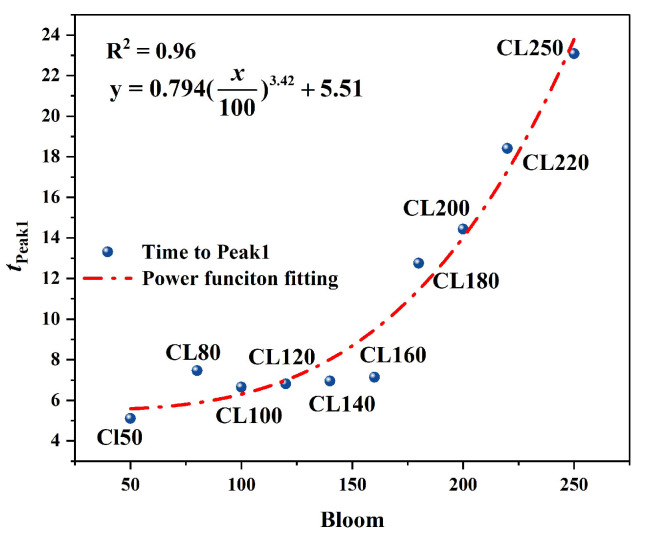
Relationship between Bloom strength and t_Peak1_ for composite gel gummy model samples measured by the CAFLT system. The solid curve shows the power regression fit; the fitted equation and R^2^ are displayed in the figure.

To quantitatively evaluate the relationship between gel strength and response time, regression analysis was performed between Bloom strength and the time corresponding to the first peak (*t*_Peak1_). Both linear and nonlinear models were examined, and the power regression provided a clearly better fit to the experimental data. Therefore, the power regression was adopted to describe this relationship (Figure 11), showing a high goodness-of-fit ($R^{2}=0.96$). The power regression reveals a monotonically increasing convex trend, suggesting that the time required to reach the first response peak grows progressively faster with increasing gel strength. As Bloom strength increased, the time to reach the first mechanical response peak became progressively prolonged, indicating that gels with higher strength require more time to reach the Hardness_CAFLT peak (Peak1) under linear airflow loading. This regression analysis further verifies the ability of the CAFLT system to discriminate samples with different hardness levels through time-domain response amplification.

Overall, by synchronously acquiring both T–AP and T–D response curves, the CAFLT system enables coordinated characterization of sample mechanical behavior in both the amplitude and time domains. The airflow pressure-domain parameter (Hardness_CAFLT) reflects resistance to deformation, whereas *t*_Peak1_ provides a time-domain descriptor that amplifies inter-sample differences. The complementary nature of these two indicators allows the proposed non-contact method to maintain good discriminative capability and detection sensitivity under small-deformation conditions.

### 5.3. Comparative Evaluation Between the CAFLT Method and Traditional TPA

In this study, the standard TPA parameters obtained using a commercial texture analyzer (TA.XT Plus) were used as the reference benchmark (control group), together with the experimentally measured Bloom strength as an independent mechanical strength indicator, to perform a multi-level statistical evaluation of the non-contact texture descriptors acquired by the CAFLT system. Pearson correlation analysis was first employed to evaluate the linear associations and comparability between the two methods at the single-parameter level. Furthermore, principal coordinates analysis (PCoA) was introduced to project the multi-parameter feature space into a lower-dimensional representation, enabling a comparison of the two methods in terms of their ability to discriminate mechanical differences among samples at the population level. Through the combined use of univariate correlations and multivariate distribution patterns, the statistical comparability, and sample discrimination capability of the non-contact CAFLT system were compared to those of traditional contact-based TPA.

Considering that the CAFLT system can simultaneously extract multiple dynamic features derived from both time–airflow pressure (T–AP) and time–displacement (T–D) curves, some parameters (e.g., T–D resilience, T–D springiness, and Time to Peak1) lack direct physical correspondence with traditional TPA definitions. Therefore, only the selected TPA-like descriptors with comparable mechanical relevance (including hardness and several response-related composite descriptors) were included in the correlation analysis, while CAFLT-specific indicators (such as T–D resilience, T–D springiness, Time to Peak1, and Pinit) were treated separately as method-specific supplementary descriptors. Because the physical meanings of some CAFLT-derived descriptors do not fully coincide with those of the corresponding traditional TPA indices, the correlation analysis was intended to assess trend-level association rather than strict one-to-one equivalence.

#### 5.3.1. Correlation Analysis of Key TPA and TPA-like Parameters

**Figure 12:**
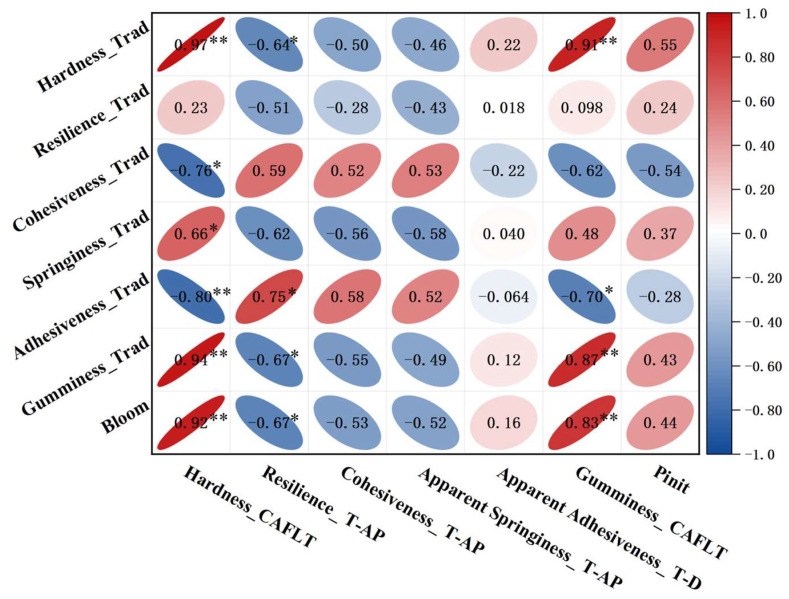
Correlation heatmap between CAFLT-derived non-contact descriptors and traditional TPA parameters, together with Bloom strength and the device-specific indicator Pinit. Pearson correlation coefficients (r) were calculated using a two-tailed test. Asterisks indicate statistical significance (* *p* < 0.05, ** *p* < 0.01). Descriptors related to adhesiveness, cohesiveness, and gumminess should be interpreted with caution because their physical meanings under CAFLT loading do not fully coincide with those of the corresponding traditional TPA parameters.

To evaluate the consistency and comparability between CAFLT-derived descriptors and the measurements obtained using the traditional contact-based probe TPA method, Pearson correlation analysis was performed on the results of ten groups of composite gel-based gummy model samples. Correlations were calculated between CAFLT-derived non-contact descriptors, traditional TPA indices, and the experimentally measured Bloom strength, and the results are presented in Figure 12. Overall, selected CAFLT-derived descriptors exhibited interpretable associations with the traditional TPA measurements, indicating that the proposed device can effectively capture major macroscopic mechanical differences among samples under non-contact and small-deformation conditions. However, the degree of comparability varied depending on the physical meaning of each parameter and the difference in loading mechanism between the two methods.

Specifically, Hardness_CAFLT showed a strong positive correlation with the traditional hardness parameter (Hardness_Trad) (r = 0.97), and both parameters exhibited highly consistent trends with Bloom strength (r = 0.92). These results demonstrate that the first peak airflow pressure extracted under airflow loading can reliably characterize the overall resistance of samples to deformation, showing strong comparability with traditional contact-based compression hardness. In addition, Gumminess_CAFLT, calculated from Hardness_CAFLT and Cohesiveness_T–AP, also exhibited a significant positive correlation with traditional gumminess (Gumminess_Trad) (r = 0.87). However, because this CAFLT-derived composite index is constructed under a different loading mechanism and involves a cohesiveness-related descriptor that does not fully share the same physical meaning as Cohesiveness_Trad, it should be interpreted as a CAFLT-derived strength-related composite indicator rather than a strict one-to-one equivalent of traditional gumminess.

Cohesiveness_T–AP showed only a moderate correlation with Cohesiveness_Trad (r = 0.52), reflecting that the two parameters are influenced by different loading pathways and response definitions. Therefore, Cohesiveness_T–AP should be interpreted as a CAFLT-derived response descriptor rather than a direct equivalent of traditional cohesiveness.

Descriptors related to Resilience_T–AP, Springiness_T–AP, and Adhesiveness_T–D showed only limited correspondence with their traditional TPA counterparts. This result is not unexpected, because these CAFLT-derived parameters are extracted from time-domain response features under pressure-programmed non-contact loading, whereas the traditional TPA indices are defined under displacement-controlled contact compression with different recovery and interfacial interaction mechanisms. Therefore, although some trend-level associations were observed for certain parameters, these descriptors should be interpreted primarily as CAFLT-specific supplementary indicators of dynamic response behavior, rather than as strict one-to-one equivalents of traditional resilience, springiness, or adhesiveness.

The device-specific parameter *P*_init_ showed generally low correlations with traditional TPA indices but a moderate positive correlation with Bloom strength (r = 0.44). This result suggests that *P*_init_ mainly reflects the initial threshold required to induce measurable deformation, rather than serving as a direct substitute for traditional TPA parameters. Accordingly, *P*_init_ is more appropriately treated as a complementary indicator of surface mechanical sensitivity.

Overall, the CAFLT method showed the strongest comparability with traditional TPA in strength-related behavior, particularly for hardness, while Gumminess_CAFLT showed a positive association with traditional gumminess and may be interpreted as a CAFLT-derived composite strength-related indicator. In contrast, descriptors related to cohesiveness, resilience, springiness, and adhesiveness showed only limited correspondence with their traditional TPA counterparts and should therefore be interpreted more cautiously as CAFLT-specific supplementary indicators of dynamic response behavior. Collectively, these findings suggest that the CAFLT system can capture major mechanical differences among samples under non-contact and nondestructive conditions, while also providing additional time-domain information beyond that obtained from traditional TPA.

Future work will focus on further optimization of the loading protocol, multivariate calibration of CAFLT-derived descriptors, and clarification of their physical meaning under non-contact small-deformation conditions. In particular, further studies are needed to better understand the mechanisms behind the observed correlation patterns and to establish more robust predictive relationships between selected CAFLT-derived indicators and benchmark texture attributes.

#### 5.3.2. Multi-Parameter Discrimination Analysis Based on PCoA

To further evaluate the comparability of non-contact CAFLT and traditional TPA measurements in terms of overall sample discrimination from a multi-parameter perspective, principal coordinates analysis (PCoA) was applied to the multi-parameter datasets obtained from both methods for distance-based ordination and visual comparison. Unlike Pearson correlation analysis, which only reflects linear relationships between individual parameters, PCoA constructs a low-dimensional ordination from a distance (dissimilarity) matrix computed using multiple texture indices, enabling the visualization of sample separation in a global multivariate feature space and facilitating an overall comparison of the discrimination patterns obtained by the two methods. Group separation in the multivariate space was further evaluated using PERMANOVA.

**Figure 13:**
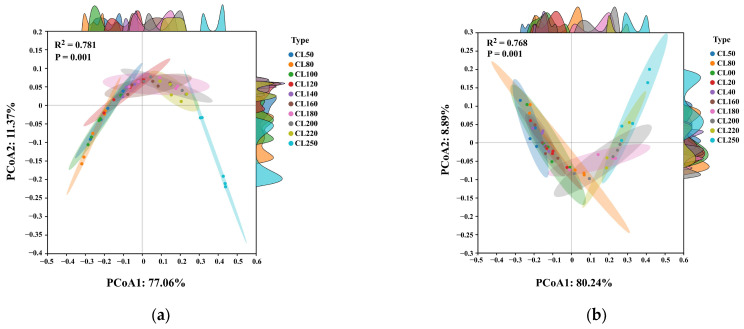
PCoA plots and circular cluster heatmaps based on texture-related descriptors obtained using two methods: (**a**) traditional TPA; (**b**) the CAFLT_TPA-like method. The R^2^ and *p* values in each panel are PERMANOVA statistics (R^2^: effect size explained by Bloom-level grouping; *p*: permutation-based significance).

Figure 13a shows the updated PCoA ordination constructed from the traditional texture analyzer parameters (TPA_trad). The first two axes (PCoA1 and PCoA2) explained 77.06% and 11.37% of the total variance, respectively (cumulative 88.43%). A clear strength-related gradient was observed mainly along PCoA1: low-strength gels (CL50–CL100) were located in the negative region, whereas high-strength gels (CL220–CL250) progressively shifted toward positive PCoA1 values, with CL250 showing the most evident endpoint separation. The marginal density distributions further indicate that the density peaks of each group shift monotonically along PCoA1 with increasing Bloom strength, confirming that gel strength dominates the multivariate structure. Intermediate groups concentrated around the apex region of the two-dimensional projection, leading to partial overlap between adjacent strength levels. PERMANOVA further confirmed significant group differences (*p* = 0.001) with a large effect size (PERMANOVA R^2^ = 0.781), indicating that Bloom grouping explains a substantial proportion of the distance-based variation in the multivariate space.

Figure 13b presents the PCoA results derived from the CAFLT_TPA-like parameter set. PCoA1 and PCoA2 explained 80.24% and 8.89% of the variance, respectively (cumulative 89.13%), which is comparable to or slightly higher than that of the traditional method. A consistent strength gradient pattern was again observed along PCoA1, with CL250 maintaining endpoint separation. Compared with TPA_trad, the CAFLT ordination exhibited a more pronounced V-shaped geometry in the 2D projection, making neighboring intermediate groups more prone to local overlap around the turning region. The marginal density plots suggest that such overlap is mainly associated with the secondary coordinate structure (PCoA2) rather than the primary strength gradient axis. Importantly, PERMANOVA still indicated significant Bloom group separation (*p* = 0.001) with a large effect size (PERMANOVA R^2^ = 0.768), close to that of the traditional method. (Note that the upright/inverted “V” appearance reflects differences in projection geometry and axis orientation rather than a direct superiority of either method.)

Overall, the two ordinations show a highly consistent global strength-related trend: samples migrate systematically with increasing Bloom strength along the principal gradient direction in the multivariate space. Although neighboring groups show a certain degree of overlap in the two-dimensional projections of both methods, PERMANOVA analysis still confirms significant separation among different Bloom levels and indicates that, under non-contact small-deformation measurements, the CAFLT method provides a similar overall pattern of multivariate discrimination to that obtained by traditional TPA.

### 5.4. Operating Envelope and Practical Limitations of the CAFLT System

In the current configuration, the CAFLT system has an operating envelope constrained by the available air pressure range (0–$P_{max}$ = 500 kPa), programmable ramp rate (dP/dt = 1 kPa/s in this study), stand-off distance ($S_{o}$ = 10 mm), laser displacement sensor working distance (75 mm) and range (55 mm), and practical test duration. A sample is considered suitable if it can reach the preset target deformation (e.g., 1.0 mm) within $P_{max}$ and the time window under the selected ramp rate, while maintaining stable surface integrity (no rupture or unstable bulging) during airflow excitation. Accordingly, the CAFLT method may have limited efficacy for (i) very stiff materials, where the target deformation cannot be reached without exceeding $P_{max}$ or an impractical duration, and (ii) extremely soft/fragile materials, where airflow excitation induces instability or rupture before a stable small-deformation response is obtained. For heterogeneous foods, the CAFLT system and probe-based TPA may sample different effective measurement footprints. Because the local loading/sensing region of CAFLT is smaller than that of a conventional TPA probe, the CAFLT response may be more sensitive to local structural heterogeneity. Therefore, standardized positioning and multi-point measurements followed by averaging are recommended to improve result representativeness. This issue will also be considered in future software development for assisted sampling and data integration.

Based on these criteria, the gelatin–maltose gel models used in this study showed stable responses under small-deformation conditions. In preliminary feasibility trials, several additional materials, including industrial gummy products, meat, and polyurethane samples, also exhibited stable waveforms and good repeatability, whereas very soft dough-skin samples tended to rupture during airflow loading, indicating a current limitation of the CAFLT system for ultra-soft thin-layer products. Under such conditions, the unloading and rebound responses became unstable, which limited reliable extraction of TPA-like descriptors. Future work will focus on expanding the operating envelope and improving robustness by optimizing protocol settings (e.g., dP/dt, target deformation, inter-cycle interval, and $S_{o}$), as well as nozzle and flow-field design, and by extending validation to a broader range of real products (including sensory evaluation where appropriate). When practical mapping to traditional TPA attributes is required for industrial implementation, multivariate calibration will be further developed as described in Section 5.3.1.

## 6. Conclusions

In this study, a non-contact texture analysis system (Controlled Airflow–Laser Texturemeter, CAFLT) integrating controlled airflow excitation with laser displacement sensing was developed to address the need for non-destructive characterization of soft food texture. By reconstructing a double-compression testing cycle through programmable airflow loading and displacement feedback control, the proposed system enabled non-contact acquisition of both stress-related and deformation-related responses. Combined with adaptive Savitzky–Golay smoothing and dual-curve feature extraction algorithms, the method effectively suppressed airflow-induced noise and improved the stability and reproducibility of small-deformation signals.

Validation using gelatin–maltose composite gel models with graded Bloom strengths demonstrated that the CAFLT results exhibited strong comparability with those from traditional contact-based TPA in strength-dominated parameters such as hardness and gumminess. Descriptors related to cohesiveness, resilience, and springiness exhibited interpretable relationships with their traditional TPA counterparts, although their physical meanings should be considered within the CAFLT-specific loading framework. Furthermore, principal coordinates analysis (PCoA) revealed comparable sample clustering patterns and mechanical gradients between the two methods, indicating comparable overall multivariate discrimination performance.

Beyond capturing the core mechanical information most directly reflected by hardness-related behavior, the CAFLT system also provided informative time-domain and dynamic descriptors, enabling enhanced differentiation of samples under small-deformation conditions. In particular, the time to the first response peak (*t*_Peak1_) showed a strong power-law relationship with Bloom strength ($R^{2}=0.96$), indicating that gels with higher strength required progressively longer response times to reach the first mechanical peak under airflow loading. This result demonstrates that the CAFLT system can amplify sample differences not only in pressure-related magnitude, but also in time-domain response behavior. Owing to its non-contact loading mechanism, the system eliminates probe–sample adhesion and structural damage, offering advantages in hygiene, repeatability, and potential for continuous online monitoring.

Overall, the proposed CAFLT system provides a feasible instrumental approach for rapid, non-destructive, and multi-parameter texture characterization of soft foods, laying the methodological foundation for real-time quality control and intelligent sensing applications in food processing. In this study, sensory evaluation was not conducted; therefore, the conclusions are limited to instrumental benchmarking against TPA_trad and Bloom strength. In addition, the CAFLT protocol was not performed under fully identical measuring conditions to those of traditional TPA_trad, because the two methods differ in loading mode, deformation scale, and rate definition. Accordingly, the present comparison should be interpreted as a benchmark-level reference for trend-level comparability and sample discrimination, rather than as strict equivalence under matched test conditions. Future work will expand to real edible products and include sensory evaluation, together with protocol optimization (e.g., pressure ramp rate, target deformation, inter-cycle interval, and stand-off distance ($S_{o}$)) and multivariate calibration to facilitate more robust predictive relationships among CAFLT descriptors, Traditional TPA attributes, and sensory perception for industrial implementation, and comparison with dynamic viscoelastic measurements under small-deformation conditions as a complementary future direction.

## Figures and Tables

**Table 2:** Measured Bloom strengths and statistical analysis of gelatin raw materials used for composite gummy model samples.

Sample ID	Nominal Bloom, g Bloom	Measured Bloom, g Bloom	Bloom Diff, g Bloom	CV, %
CL_50	50	36.27 ± 2.76	−13.73	7.61%
CL_80	80	40.70 ± 1.07	−39.30	2.63%
CL_100	100	47.26 ± 0.43	−52.74	0.91%
CL_120	120	61.00 ± 1.86	−59.00	3.05%
CL_140	140	103.26 ± 2.79	−36.74	2.70%
CL_160	160	111.32 ± 1.24	−48.68	1.11%
CL_180	180	124.03 ± 1.63	−55.97	1.31%
CL_200	200	132.80 ± 0.44	−67.20	0.33%
CL_220	220	197.61 ± 2.05	−22.39	1.04%
CL_250	250	258.30 ± 0.58	8.30	0.22%
Average			−38.75	2.09%

**Table 3:** TPA test program parameters of the texture analyzer.

Program Setting	Settings and Values
Test mode	TPA
Pre-test Speed	1 mm/s
Test Speed	0.50 mm/s
Post-test Speed	0.50 mm/s
Strain	75%
Trigger Mode	Auto
Trigger Force	5.0 g
Probe Type	P/0.5 DIA DELRIN AOAC FOR GELATINE

**Table 4:** Statistics of data validity and SNR changes before and after LOF denoising.

Samples	Original Valid Data Ratio	Valid Data Ratio After Processing	Original Signal Smoothness	Processed Signal Smoothness	Signal-to-Noise Ratio (SNR)
CL_50 Means	0.868	0.850	0.072	0.068	118.7
CL_80 Means	0.790	0.774	0.085	0.075	29.9
CL_100 Means	0.844	0.827	0.080	0.069	97.3
CL_120 Means	0.814	0.797	0.104	0.086	220.9
CL_140 Means	0.864	0.846	0.099	0.084	64.6
CL_160 Means	0.836	0.819	0.088	0.076	106.3
CL_180 Means	0.818	0.802	0.204	0.188	8.4
CL_200 Means	0.774	0.758	0.111	0.103	22.0
CL_220 Means	0.839	0.822	0.097	0.091	41.7
CL_250 Means	0.576	0.565	0.054	0.052	83.3

Note: The signal-to-noise ratio (SNR) was calculated as the ratio between the root-mean-square (RMS) amplitude of the processed displacement signal and that of the residual high-frequency noise component obtained by subtracting the smoothed signal from the raw time–displacement signal.

**Table 5:** Comparison of error metrics between fixed-window and adaptive-window SG smoothing.

Sample	Fixed-Window Size	*DP*1	*DP*2	Peak Diff	*E_combined_*	Adaptive- Window Size	*DP*1	*DP*2	Peak Diff	*E_combined_*
CL_50 Means	17	1.0827	0.9591	0.1416	0.2429	23	1.0427	0.9539	0.0922	0.1731
CL_80 Means	17	0.9909	0.9042	0.1696	0.3229	31	0.9261	0.8602	0.0838	0.2080
CL_100 Means	17	0.9395	1.0353	0.1842	0.2899	25	0.9304	1.0193	0.1294	0.2026
CL_120 Means	17	0.9044	0.8700	0.1316	0.2564	26	0.8815	0.8483	0.0861	0.2246
CL_140 Means	17	0.8469	0.8951	0.0770	0.2060	20	0.8704	0.8874	0.0283	0.1568
CL_160 Means	17	0.9459	0.9229	0.0453	0.1149	15	0.9702	0.9574	0.0242	0.0606
CL_180 Means	17	0.9455	0.8869	0.2321	0.4155	19	0.9107	0.9395	0.0752	0.1801
CL_200 Means	17	0.9638	0.9351	0.0805	0.1555	18	0.9188	0.9442	0.0450	0.1137
CL_220 Means	17	0.9080	0.8641	0.0644	0.1867	20	0.9078	0.8811	0.0330	0.1386
CL_250 Means	17	0.9493	0.9310	0.0257	0.0857	21	0.9586	0.9464	0.0189	0.0664

**Table 6:** Summary of characteristic parameters of composite gel gummy samples obtained by the CAFLT system.

Sample/ (Mean ± SD)	*P*_init_ (kPa)	Hardness_CAFLT (kPa)	Time to Peak1_T-AP (s)	Resilience_T-AP	Cohesiveness_T-AP	Apparent Springiness_T-AP	Apparent Adhesiveness_T-D (mm·s)	Resilience_T-D	Springiness _ T-D	Gumminess_CAFLT (kPa)
CL50	3.37 ± 0.25	4.53 ± 0.81	4.53 ± 0.81	1.95 ± 0.55	1.18 ± 0.33	1.33 ± 0.44	0.14 ± 1.40	2.65 ± 0.85	1.50 ± 1.09	5.27 ± 1.35
CL80	5.47 ± 0.98	7.47 ± 3.40	7.46 ± 3.40	1.68 ± 1.63	0.98 ± 0.25	1.20 ± 0.50	1.04 ± 0.10	2.28 ± 2.37	2.24 ± 1.93	6.90 ± 1.44
CL100	4.92 ± 2.46	6.80 ± 0.17	6.80 ± 0.17	1.24 ± 0.62	1.22 ± 0.13	1.13 ± 0.27	0.12 ± 0.05	1.25 ± 0.51	1.55 ± 0.46	8.31 ± 0.71
CL120	2.98 ± 0.23	7.37 ± 0.95	7.37 ± 0.95	1.35 ± 0.38	0.92 ± 0.20	1.15 ± 0.51	0.04 ± 0.03	2.18 ± 0.86	1.26 ± 0.38	6.68 ± 1.12
CL140	4.74 ± 0.35	6.63 ± 0.60	6.64 ± 0.60	1.84 ± 1.00	1.17 ± 0.22	1.51 ± 0.05	0.12 ± 0.01	2.87 ± 3.01	0.94 ± 0.19	7.76 ± 1.49
CL160	3.81 ± 0.11	7.15 ± 0.59	6.72 ± 0.60	1.07 ± 0.19	0.94 ± 0.05	0.77 ± 0.27	0.08 ± 0.01	1.64 ± 0.47	0.76 ± 0.33	3.37 ± 3.94
CL180	5.57 ± 1.77	15.08 ± 2.40	15.08 ± 2.40	1.08 ± 0.48	0.71 ± 0.36	0.78 ± 0.35	0.25 ± 0.18	1.32 ± 0.56	0.77 ± 0.40	10.10 ± 3.69
CL200	7.38 ± 0.09	16.51 ± 1.05	16.51 ± 1.05	0.95 ± 0.01	0.81 ± 0.18	0.80 ± 0.09	0.39 ± 0.12	1.05 ± 0.05	0.81 ± 0.09	13.29 ± 2.12
CL220	1.87 ± 1.53	17.24 ± 2.86	17.23 ± 2.86	0.85 ± 0.07	0.65 ± 0.33	0.65 ± 0.31	0.08 ± 0.06	1.18 ± 0.30	0.72 ± 0.36	11.75 ± 7.57
CL250	9.31 ± 8.34	27.66 ± 5.40	27.66 ± 5.40	1.00 ± 0.08	0.96 ± 0.13	1.02 ± 0.26	0.76 ± 0.68	1.18 ± 0.11	0.99 ± 0.22	26.89 ± 8.43

## Data Availability

The original contributions presented in this study are included in the article/Supplementary Materials. Further inquiries can be directed to the corresponding author.

## Supplementary Materials

The following supporting information can be downloaded at: https://www.mdpi.com/article/10.3390/foods15071166/s1, Figure S1. Steady-state CFD contours of total pressure on the nozzle outlet plane under different inlet set pressures. Figure S2. Linear regression between the inlet set pressure $P_{in}$ the nozzle outlet-plane area-weighted mean total pressure ${\bar{P}}_{\mathrm{out},\mathrm{tot}}$ obtained from steady-state CFD simulations. Figure S3. Relationship between nominal and measured Bloom strengths of the gelatin raw materials.

## Abbreviations

The following abbreviations are used in this manuscript:

TPA	Texture Profile Analysis
CAFLT	Controlled Airflow–Laser Texturemeter
T-AP	Time–Airflow Pressure
T–D	Time–Displacement
SG	Savitzky–Golay
LOF	Local Outlier Factor
PCoA	Principal Coordinates Analysis
SNR	Signal-to-Noise Ratio
