# Supplementary material for: Non-Contact Characterization of TPA-like Texture Properties of Gel-Based Soft Foods Using a Controlled Airflow–Laser System
Source: Foods. 2026 Mar 30;15(7):1166. doi: 10.3390/foods15071166 (PMC13073278; doi:10.3390/foods15071166)
Supplement: Supplementary file 1 [file foods-15-01166-s001.zip › foods-4168988-supplementary.pdf]

Supplementary Materials

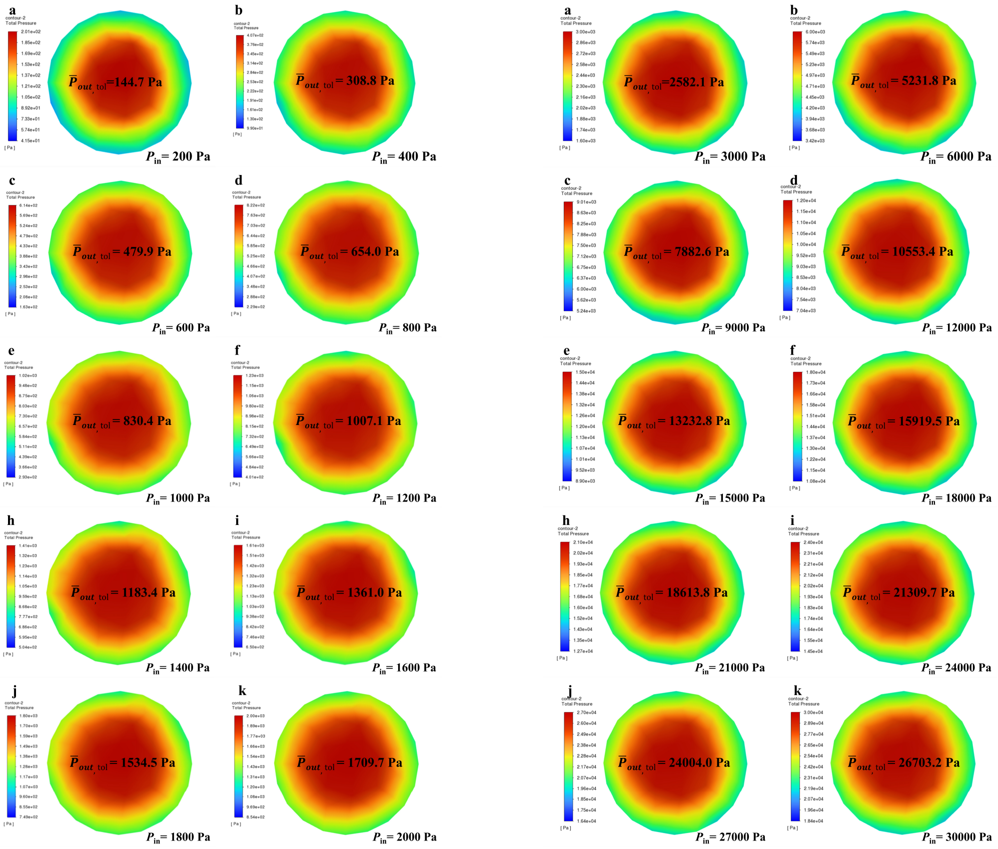

(**a**) (**b**)

Figure S1. Steady-state CFD contours of total pressure on the nozzle outlet plane under different inlet set pressures. (**a**) Low-pressure regime (200–2000 Pa); (**b**) high-pressure regime (3000–30000 Pa), corresponding to the pressure range relevant to the CAFLT experiments. Panels (a–k) represent increasing inlet set pressures (P_{in}). The value shown at the center of each panel is the outlet-plane area-weighted mean total pressure ($P_{out,tot}$), obtained from the CFD surface report. The color scale is auto-adjusted for each panel to highlight spatial distribution; therefore, quantitative comparison across inlet pressures is based on the reported mean values and the regression results shown in Figure S2.

 https://doi.org/10.3390/foods15071166

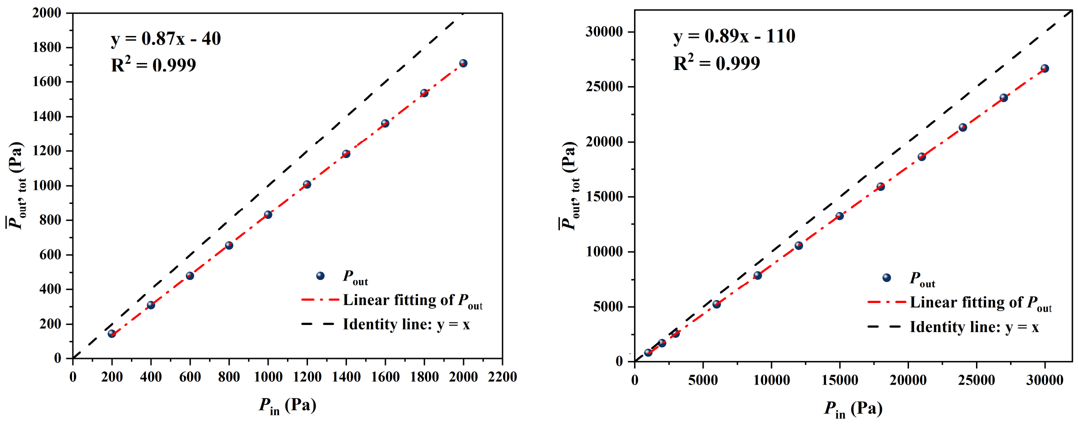

(**a**) (**b**)

Figure S2. Linear regression between the inlet set pressure P_{in} and the nozzle outlet-plane area-weighted mean total pressure $\bar{P}_{\text{out,tot}}$ obtained from steady-state CFD simulations. (**a**) Low-pressure regime (200–2000 Pa); (**b**) High-pressure regime (3000–30000 Pa), which is comparable to the pressure range covered in the CAFLT experiments. Symbols represent CFD-extracted mean values, and the red dashed line shows the linear fit; the fitted equation and $R^2$are reported in each panel. The black dashed line denotes the identity line (y = x).

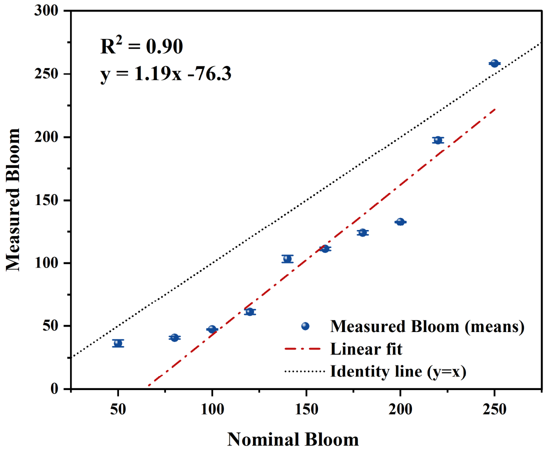

Figure S3. Relationship between nominal and measured Bloom strengths of the gelatin raw materials. Symbols represent the mean measured Bloom strength for each nominal grade (n = 5), and error bars indicate ±SD. The black dotted line denotes the identity line (y = x), and the red dashed line shows the linear regression based on the grade means (N = 10).
